# LlWRKY39 is involved in thermotolerance by activating *LlMBF1c* and interacting with LlCaM3 in lily (*Lilium longiflorum*)

**DOI:** 10.1038/s41438-021-00473-7

**Published:** 2021-02-04

**Authors:** Liping Ding, Ze Wu, Renda Teng, Sujuan Xu, Xing Cao, Guozhen Yuan, Dehua Zhang, Nianjun Teng

**Affiliations:** 1 Key Laboratory of Landscaping Agriculture, Ministry of Agriculture and Rural Affairs, College of Horticulture, Nanjing Agricultural University, Nanjing, 210095 China; 2 Baguazhou Science and Technology Innovation Center of Modern Horticulture Industry, Nanjing, 210043 China; 3 College of Agriculture, Nanjing Agricultural University, Nanjing, 210095 China; 4 State Key Laboratory of Microbial Technology, Shandong University, Qingdao, 266237 China; 5 College of Life Science, Zaozhuang University, Zaozhuang, 277160 China

**Keywords:** Plant sciences, Plant stress responses, Heat

## Abstract

WRKY transcription factors (TFs) are of great importance in plant responses to different abiotic stresses. However, research on their roles in the regulation of thermotolerance remains limited. Here, we investigated the function of LlWRKY39 in the thermotolerance of lily (*Lilium longiflorum* ‘white heaven’). According to multiple alignment analyses, LlWRKY39 is in the WRKY IId subclass and contains a potential calmodulin (CaM)-binding domain. Further analysis has shown that LlCaM3 interacts with LlWRKY39 by binding to its CaM-binding domain, and this interaction depends on Ca^2+^. *LlWRKY39* was induced by heat stress (HS), and the LlWRKY39-GFP fusion protein was detected in the nucleus. The thermotolerance of lily and Arabidopsis was increased with the ectopic overexpression of *LlWRKY39*. The expression of heat-related genes *AtHSFA1*, *AtHSFA2*, *AtMBF1c*, *AtGolS1*, *AtDREB2A*, *AtWRKY39*, and *AtHSP101* was significantly elevated in transgenic Arabidopsis lines, which might have promoted an increase in thermotolerance. Then, the promoter of *LlMBF1c* was isolated from lily, and LlWRKY39 was found to bind to the conserved W-box element in its promoter to activate its activity, suggesting that LlWRKY39 is an upstream regulator of *LlMBF1c*. In addition, a dual-luciferase reporter assay showed that via protein interaction, LlCaM3 negatively affected LlWRKY39 in the transcriptional activation of *LlMBF1c*, which might be an important feedback regulation pathway to balance the LlWRKY39-mediated heat stress response (HSR). Collectively, these results imply that LlWRKY39 might participate in the HSR as an important regulator through Ca^2+^-CaM and multiprotein bridging factor pathways.

## Introduction

High temperature is one of the unfavorable factors affecting the growth of plants, generally impairing photosynthetic activity and negatively affecting cell division and growth^1^. Extreme high temperatures may result in a series of morphoanatomical and physiochemical changes in plant cells and even lead to severe economic losses in crops and other economically important plants^2,3^. Plants must produce various defense mechanisms against high temperature, including the accumulation of heat shock proteins (HSPs) and complex regulatory networks as established by transcription factors (TFs)^4,5^.

Lily (*Lilium* spp.) is one of the most popular cut flower products worldwide because of its attractive shape and color^6^. Lily adapts well to cool conditions but is sensitive to high temperatures (>30 °C), which not only reduces the quality of cut flowers but also leads to the degeneration of the bulb^7^. However, high temperatures will become an unavoidable environmental stress factor in the future because of the irreversible trend in global warming^8,9^. Therefore, an understanding of the HSR mechanisms of lily under HS is essential to improve the thermotolerance of lily.

TFs play major roles in increasing the stress tolerance of plants since they can regulate critical downstream genes by binding to *cis*-elements in gene promoters^10,11^. In the HSR, HS transcription factors (HSFs) can directly regulate the expression of downstream genes by binding to HS elements (HSEs; nGAAnnTTCn) in the promoters of downstream genes in response to HS^12,13^. Currently, most studies on thermotolerance in lily focus on HSFs. The overexpression of *LlHSFA1* and *LlHSFA2* from lily in Arabidopsis can enhance the thermotolerance of transgenic lines^6,7,14^. Two *HSFA3* homologs of lily, *LlHSFA3A* and *LlHSFA3B*, increase the thermotolerance of transgenic Arabidopsis plants, possibly through a proline-mediated pathway^15^. In addition, LlHSFA3A and LlHSFA3B from lily can form a regulatory mechanism involving heat-inducible alternative splicing to sustain balance in the HSR^16^. Furthermore, lily LlDREB2B, a member of the DREB subfamily of the ERF/AP2 TF family, can increase the basal thermotolerance (BT) and acquired thermotolerance (AT) of transgenic Arabidopsis^17^.

Multiprotein bridging factor 1 (MBF1) is a highly conserved transcriptional coactivator with various forms involved in the regulation of diverse processes, such as oxidative stress, hormone-regulated seed germination, and translation^18–20^. In Arabidopsis, MBF1 cofactors are encoded by three genes: *AtMBF1a* (*AT2G42680*), *AtMBF1b* (*AT3G58680*), and *AtMBF1c* (*AT3G24500*)^21^. Among these genes, *AtMBF1c* is related to thermotolerance and functions upstream of salicylic acid (SA), ethylene, and trehalose signaling^22,23^. A previous study reported that *AtMBF1c* is regulated by AtHsfA1 cofactors because an *hsfa1* quadruple mutant shows suppressed expression of *AtMBF1c* during HS^24^. Despite much information available indicating how plant *MBF1c* genes respond to HS, many questions about the relationship between *MBF1c* and other important TFs, e.g., WRKY TFs, remain.

WRKY TFs can participate in multiple adverse responses during plant growth and development^25–27^. The typical feature of WRKY TFs is the WRKY domain, which contains an invariant WRKYGQK sequence and a zinc finger motif (CX_4–5_CX_22–23_HXH or CX_7_CX_23_HXC)^28,29^. The WRKY superfamily is classified into three groups based on the number of conserved WRKY signatures (two WRKY sequences in group I and one WRKY sequence in groups II and III) and the composition of the zinc finger motif in which the zinc finger motif of groups I and II is CX_4–5_CX_22–23_HXH and the zinc finger motif of group III is CX_7_CX_23_HXC^22^. Group II is further classified into five subgroups (IIa to IIe) based on different conserved short motifs^22,28^. WRKY TFs can recognize and bind to W-box elements (TTGACC/T) in the promoters of resistance-related genes. The core sequence TGAC is essential for WRKY recognition, as indicated by different binding experiments^30,31^. This type of binding can regulate the expression of target stress genes and thus increase plant stress tolerance^29,32^.

In recent years, increasing evidence has shown that WRKYs are related to thermotolerance^33–36^. However, studies on WRKY TFs are primarily focused on crops or model plants such as rice, wheat, and Arabidopsis, and little research has been carried out on lily. In *A. thaliana*, the group II WRKY protein AtWRKY39 may play a positive role in thermotolerance by regulating the cooperation between the SA- and JA-activated signaling pathways^34^. Here, we identify a WRKY-IId factor in lily, LlWRKY39, which was induced by HS and can interact with LlCaM3 in a Ca^2+^-dependent manner. The thermotolerance of transgenic plants increased with the overexpression of *LlWRKY39*. Further analysis indicated that LlWRKY39 can bind to the promoter of *LlMBF1c* and activate its expression. However, the interaction between LlCaM3 and LlWRKY39 negatively affected the transactivation of *LlMBF1c* induced by LlWRKY39, which may imply feedback regulation of LlWRKY39 to maintain a balance during the HSR. The results of this study may help to reveal the biological function and mechanism of LlWRKY39 under HS and provide an important theoretical foundation for further perfecting the HS signal transduction network regulated by lily TFs.

## Results

### LlWRKY39 is a heat-inducible member of the WRKY group IId family of transcription factors

**Fig. 1:**
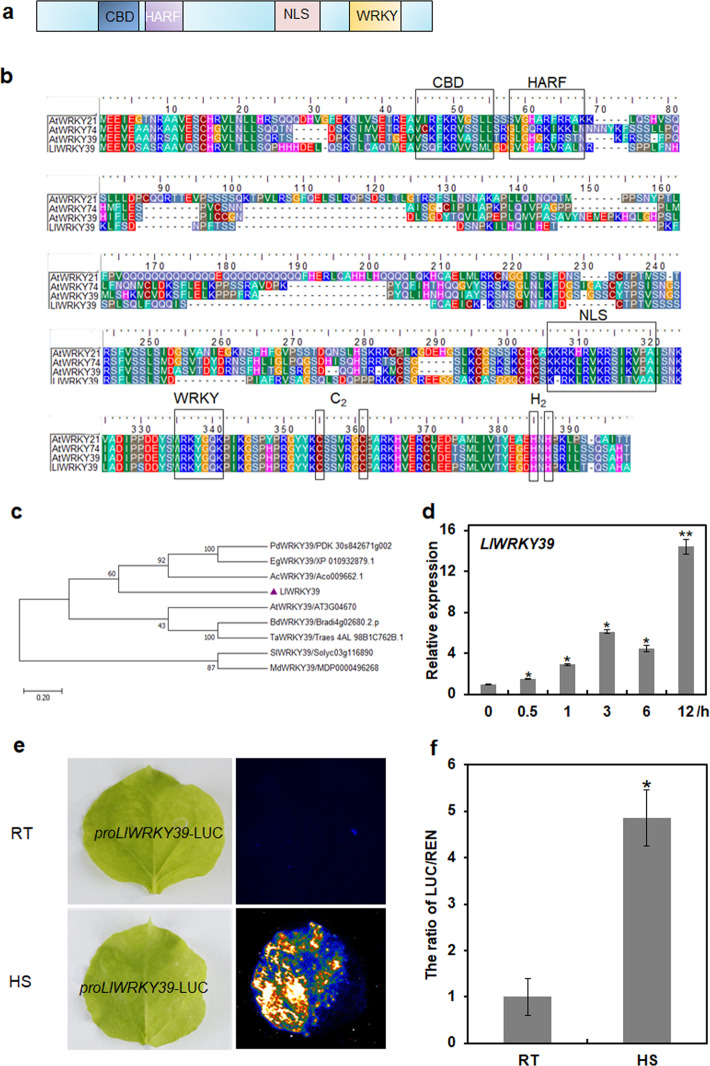
*LlWRKY39* is a heat-inducible member of the WRKY group IId of transcription factors. **a** Structural diagram of the WRKY IId transcription factor. **b** Multiple alignments of LlWRKY39 with AtWRKY39 (AT3G04670), AtWRKY74 (AT5G28650), and AtWRKY21 (AT2G30590) using the Clustal W algorithm with default parameters. The approximate 60-amino acid WRKY domain, CaM-binding domain (CBD), HARF motif, and nuclear localization site (NLS) are indicated by black boxes. **c** Phylogenetic relationship of LlWRKY39 with other plant WRKY proteins was determined by the neighbor-joining method with 500 bootstrap replicates. LlWRKY39 is highlighted with a purple triangle. The following abbreviations are used to indicate the scientific names of plants with WRKY: AC ananas comosus, EG elaeis guineensis, PD phoenix dactylifera, BD brachypodium distachyon, TA triticum aestivum, AT arabidopsis thaliana, MD malus domestics, Sl solanum lycopersicum. **d** Relative expression of *LlWRKY39* in lily leaves under HS for different lengths of time. Each bar indicates the mean ± SD of three repeated experiments (*P < 0.05 and **P < 0.01, Student’s *t* test). **e** Analysis of *t*he promoter activity in *N. benthamiana* leaves under room temperature (RT) and HS (37 °C 2 h). **f** The relative value of LUC/REN. The ratio of LUC/REN at RT was set to 1 for normalization. All the values represent the mean ± SD of three repeated experiments (*P < 0.05, Student’s *t* test)

We searched *AtWRKY39* from the Arabidopsis database with the online TAIR tool (https://www.arabidopsis.org/) and selected putative WRKY39 from the pollen transcriptome database of ‘little kiss’^37^. Then, we cloned putative WRKY39 from ‘white heaven’. Thus, the candidate was designated LlWRKY39. The full-length cDNA sequence of *LlWRKY39* contains an 858-bp open reading frame (ORF) that encodes a 285-amino acid protein. The phylogenetic tree including all WRKY proteins in Arabidopsis showed that LlWRKY39 is closely related to AtWRKY39, AtWRKY74, and AtWRKY21 (Supplementary Fig. S1), which suggests that LlWRKY39 is a member of the WRKY group IId family. To identify the basic characteristics of LlWRKY39, amino acid sequence alignment of LlWRKY39, AtWRKY39, AtWRKY74, and AtWRKY21 was performed. The sequences exhibited a similar signature, which included a WRKYGQK sequence, one zinc-binding motif C–X_5_–C–X_23_–H–X_1_–H, one HARF (RTGHARFRR[A/G]P) motif, a nuclear localization site (NLS), and a potential calmodulin (CaM)-binding domain (CBD) (Fig. 1b). Then, we investigated the evolutionary relationship between LlWRKY39 and WRKY39 factors in different plants, including Arabidopsis, tomato, apple, wheat, brachypodium, date, oil palm, and pineapple. The phylogenetic results indicated that the closest relationship was established between LlWRKY39 and pineapple AcWRKY39 (Fig. 1c), which are both noncereal monocot species.

*LlWRKY39* can be rapidly induced under HS at 37 °C for 0.5 h, and following prolonged HS for 12 h, the expression of *LlWRKY39* is enhanced significantly (Fig. 1d). The expression results suggested that *LlWRKY39* is induced by high temperature over a long period. To further examine the underlying mechanism of *LlWRKY39* under HS, we isolated and analyzed the promoter of *LlWRKY39*. Different kinds of *cis*-elements were found in the promoter of *LlWRKY39*, such as light-responsive elements (I-BOX and GATA-BOX), an element for pollen-specific expression (POLLEN1), a binding site for WRKY TFs (W-box), CCAAT-BOX, and others, indicating that *LlWRKY39* may be regulated by different types of TFs (Supplementary Table S1). Among these motifs, CCAAT-BOX is related to HS, and it can act cooperatively with HSEs to increase HS promoter activity^38^. To identify the promoter activity of *LlWRKY39* under HS, we conducted a transient luciferase reporter assay with *Nicotiana benthamiana* leaves. The LUC signal increased profoundly after subjection to HS, although under normal conditions, the LUC signal was very low, which implied that HS activated the promoter activity of *LlWRKY39* (Fig. 1e, f). Thus, these results indicate that *LlWRKY39* is a heat-inducible WRKY group IId factor.

### LlCaM3 interacts with LlWRKY39 by binding the CBD

**Fig. 2:**
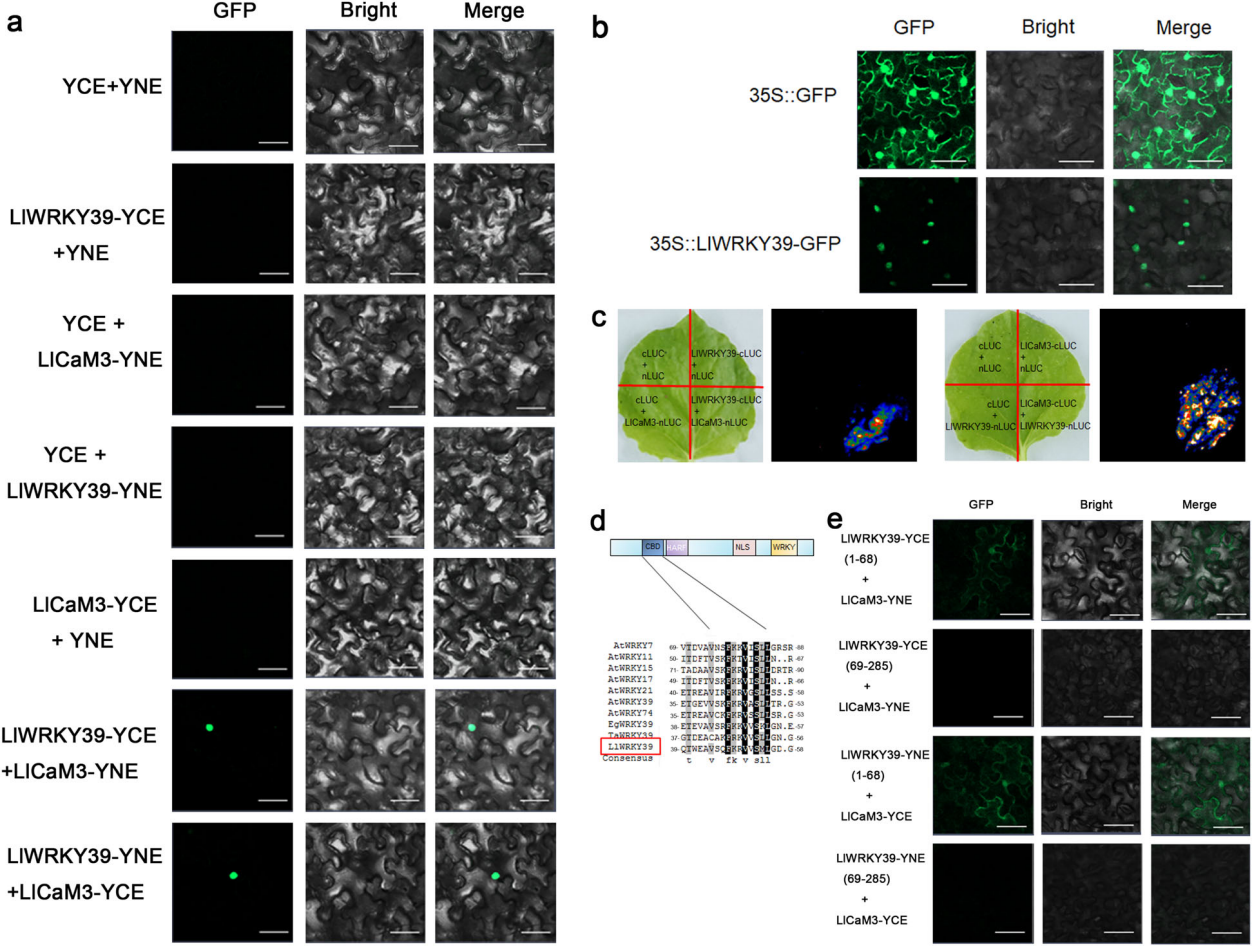
Interaction between LlWRKY39 and LlCaM3 by binding the CaM-binding domain. **a** BIFC assay. Fluorescence signals were observed using a confocal microscope. Bars = 50 μm. **b** Transient expression of *LlWRKY39* in *N. benthamiana* leaves. Bar = 50 μm. **c** FLC assay. LUC signals were observed using a CCD camera. **d** Alignment of the CBD among WRKY group IId members in Arabidopsis, oil palm, wheat, and lily. Identical amino acids are shaded in black and gray. **e** Interaction of the CBD of LlWRKY39 and LlCaM3 as determined by BIFC. Bar = 50 μm

In the protein sequence assay, a potential CBD was found in LlWRKY39 (Fig. 1b), which suggests that LlWRKY39 is a CaM-binding protein (CBP). Therefore, we evaluated whether LlWRKY39 interacted with a heat-inducible CaM, LlCaM3, from lily (Supplementary Fig. S2). Bimolecular fluorescence complementation (BIFC) showed that the fluorescence generated upon LlWRKY39 interacting with LlCaM3 was emitted only in the nucleus (Fig. 2a). A previous study showed that LlCaM3 is a cytoplasmic and nuclear protein^39^. Here, we found that the fluorescence of the LlWRKY39-GFP fusion protein appeared only in the nucleus (Fig. 2b), which might explain why the fluorescence emitted by LlWRKY39 interacting with LlCaM3 appeared only in the nucleus. Similarly, firefly luciferase complementation imaging (FLC) assays also showed that LlWRKY39 interacted with LlCaM3 (Fig. 2c). However, compared with that of homologous genes, the CBD of LlWRKY39 is not highly conserved (Fig. 2d). Therefore, we sought to determine whether the interaction between LlWRKY39 and LlCaM3 depends on this domain. We truncated LlWRKY39 into two parts, one segment with the CBD and one without the CBD. In the BIFC assay, LlWRKY39 with the CBD interacted with LlCaM3, but LlWRKY39 lacking the CBD did not (Fig. 2e). Notably, the interaction signal was observed throughout the cytoplasm and nucleus, which may be explained by the deletion of the NLS of LlWRKY39 and LlCaM3 in the cytoplasm and nucleus^30^. In a split-ubiquitin assay with yeast cells, similar results were observed (Supplementary Fig. S3), which implies that the interaction between LlCaM3 and LlWRKY39 depends on the CBD region.

### LlCaM3–LlWRKY39 interaction depends on Ca^2+^

**Fig. 3:**
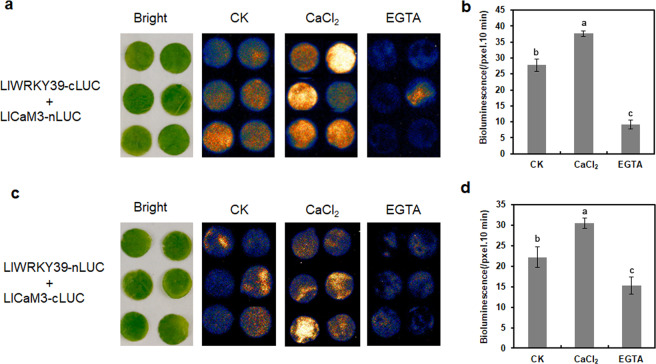
Effects of CaCl_2_ and EGTA treatment on the interaction between LlWRKY39 and LlCaM3 in *N. benthamiana* leaves. **a** LUC bioluminescence in *N. benthamiana* leaves coinfiltrated with mixed bacterial solutions of LlWRKY39-cLUC and LlCaM3-nLUC by different treatments. **b**, **d** LUC bioluminescence intensity was quantified using Andor Solis v15 software. **c** LUC bioluminescence in *N. benthamiana* leaves coinfiltrated with mixed bacterial solutions of LlWRKY39-nLUC and LlCaM3-cLUC by different treatments. Values represent the mean ± SD of three independent experiments. Significant differences are indicated by Student–Newman–Keuls test

To determine whether the LlCaM3–LlWRKY39 interaction depended on Ca^2+^, we conducted a transient FLC assay with *N. benthamiana* leaves and CaCl_2_ and calcium ion chelator ethylene glycol tetraacetic acid (EGTA) treatments. The interaction signal in the leaves treated with CaCl_2_ was stronger than that in the leaves treated with water, but the interaction signal was significantly repressed in the leaves treated with EGTA, which implies that the LlCaM3–LlWRKY39 interaction might depend on Ca^2+^ (Fig. 3).

### Overexpression of *LlWRKY39* increases the thermotolerance of transgenic plants

**Fig. 4:**
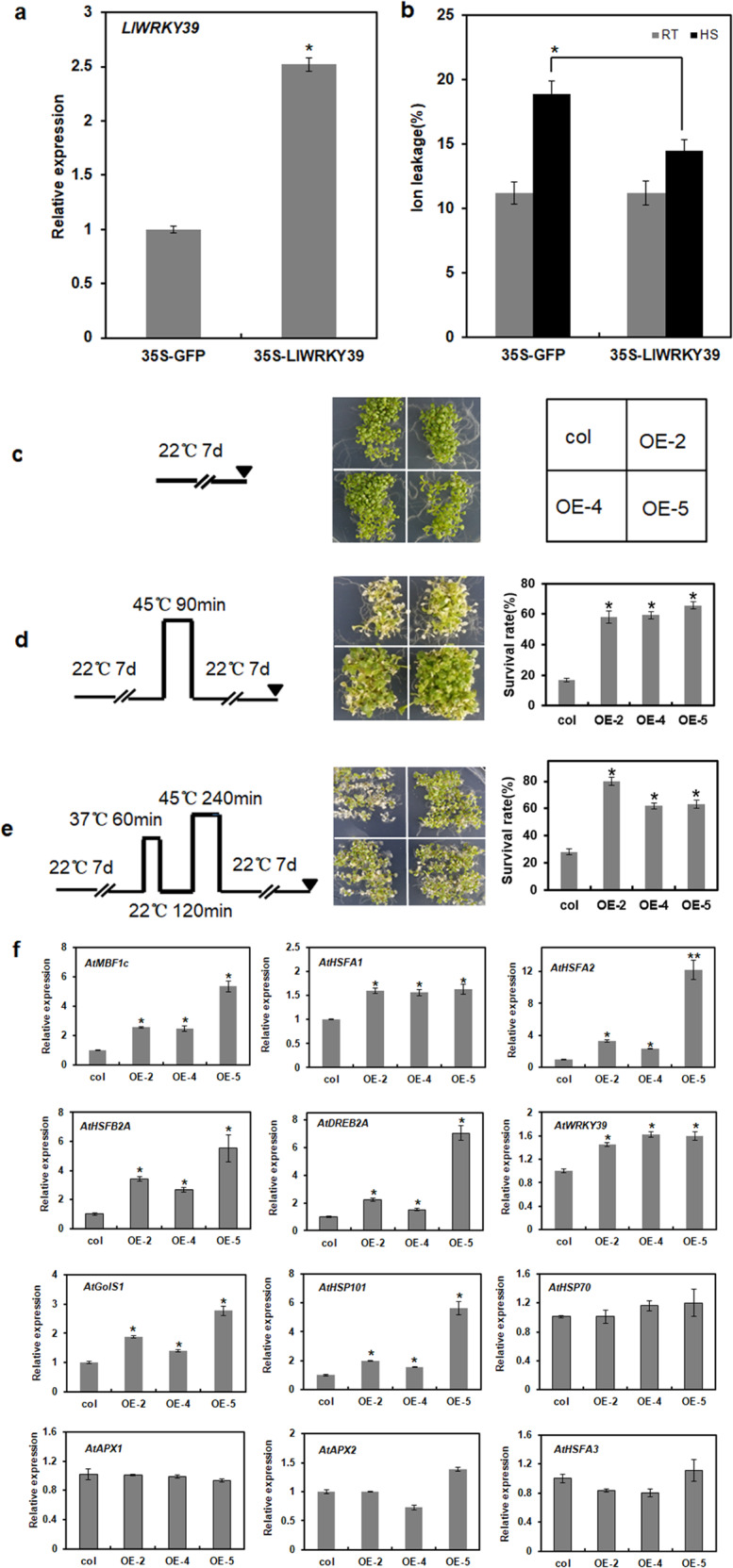
Thermotolerance assay in lily and transgenic Arabidopsis lines. **a** The expression of *LlWRKY39* in transient-overexpressing lily. **b** Relative ion leakage (%) of lily leaves under RT and HS (42 °C, 2 h). Each bar indicates the mean ± SD of three repeated experiments (*t* test, *P < 0.05). **c** Phenotypes of one-week-old seedlings before exposure to HS. **d** Phenotypes of one-week-old seedlings after the BT test. left: schematic representations of BT; right: survival rate of BT. **e** Phenotypes of one-week-old seedlings after the AT test. left: schematic representations of AT; right: survival rate of AT. A bar shows the mean of three independent experiments (*P < 0.05, *t* test). **f** Relative expression levels of HS-inducible genes in the transgenic plants. Each bar indicates the mean ± SD of three repeated experiments. Significant differences are indicated by *t* test (*P < 0.05 and **P < 0.01)

In a transient expression assay, the overexpression of *LlWRKY39* in lily leaves did not affect their relative ion leakage under normal growth conditions. However, the relative ion leakage of ‘white heaven’ leaves in the control was significantly higher than that of leaves transformed with *LlWRKY39* under HS, indicating that the transient expression of *LlWRKY39* can protect the cells of lily from high temperature (Fig. 4a, b). Four homozygous T3 transgenic Arabidopsis lines were identified (Supplementary Fig. S4), and three transgenic Arabidopsis lines (OE-2, OE-4, and OE-5) were selected for BT and AT analyses. The survival rate of the WT plants was significantly lower than that of the three transgenic lines under both HS conditions (Fig. 4d, e), which showed that the overexpression of *LlWRKY39* can increase the BT and AT of transgenic Arabidopsis. In addition, the expression levels of HS-inducible genes *AtHSFA1*, *AtHSFA2*, *AtHSP101*, *AtDREB2A*, *AtMBF1c*, *AtGolS1*, and *AtWRKY39* increased significantly in transgenic Arabidopsis (Fig. 4f), which might facilitate the increase in thermotolerance of transgenic lines. Unexpectedly, the expression of *AtHSFB2A*, a transcriptional repressor^40^, was also significantly induced. In addition, the expression of *AtHSP70*, *AtAPX1*, *AtAPX2*, and *AtHSFA3*, which are the downstream genes of *AtHSFA1*, *AtHSFA2*, and *AtDREB2A*^41–43^, did not change significantly, suggesting that LlWRKY39 also participated in other pathways to control the expression of these genes. We also measured the expression level of genes such as *LlHSFA1*, *LlHSFA2*, and *LlDREB2B* in transiently overexpressed *LlWRKY39* lily leaves. The qRT-PCR results showed that the expression levels of the *LlHSFA1*, *LlHSFA2*, and *LlDREB2B* genes were enhanced in lily leaves overexpressing *LlWRKY39* (Supplementary Fig. S5).

### LlWRKY39 activates the expression of *LlMBF1c*

**Fig. 5:**
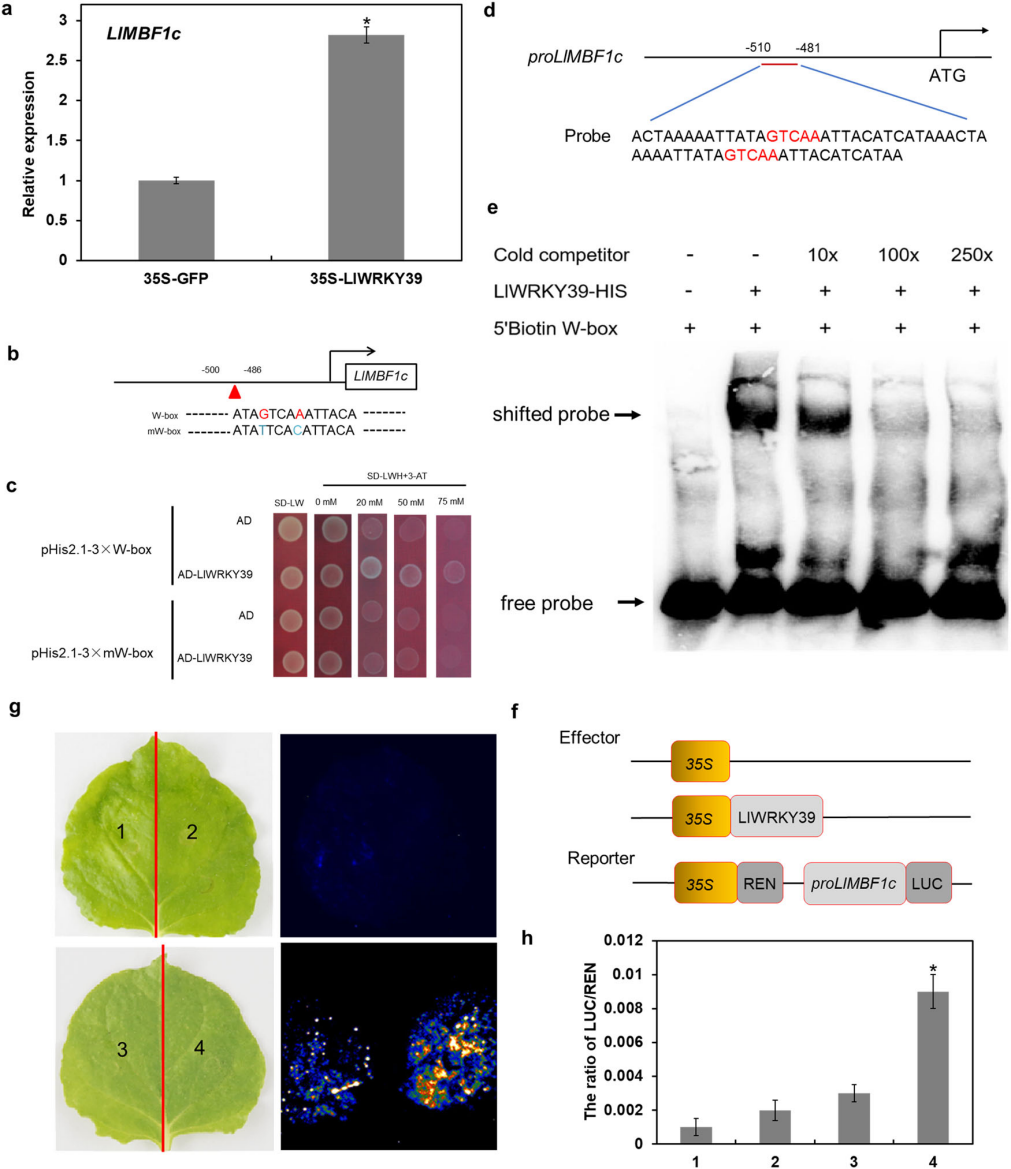
LlWRKY39 activates the expression of *LlMBF1c*. **a** Transient expression of *LlWRKY39* in lily increased the expression of *LlMBF1c*. Each bar indicates the mean ± SD of three repeated experiments. Significant differences between the control and transient overexpression plants were determined by t test (*P < 0.05). **b** The sequence of the *LlMBF1c* promoter and its W-box mutated version; the red label indicates the W-box element. **c** Yeast one-hybrid assay. The interaction between LlWRKY39 and W-box elements was determined in SD-LWH medium with different concentrations of 3-AT. **d** Schematic diagram of the *LlMBF1c* promoter; the probe sequence is shown below the diagram. **e** LlWRKY39-HIS complex binds to the W-box in EMSA. The term 250× indicates the usage of excess unlabeled probe as a competitor, and “+” and “−” indicate its presence and absence, respectively. **f** The schematic diagram of the effector and reporter. The 836-bp fragment of the *LlMBF1c* promoter was used in this assay. **g** Bright field and dark field images of *N. benthamiana* leaves in the transient expression assays. **h** The ratio of LUC/REN. 1: mixed bacterial solutions of pGreenII-62-SK and pGreenII-0800-LUC (2:1); 2: mixed bacterial solutions of pGreenII-62-SK-LlWRKY39 and pGreenII-0800-LUC (2:1); 3: mixed bacterial solutions of pGreenII-62-SK and pGreenII-0800-*proLlMBF1c*-LUC (2:1); 4: mixed bacterial solutions of pGreenII-62-SK-LlWRKY39 and pGreenII-0800-*proLlMBF1c*-LUC (2:1). Each bar indicates the mean ± SD of three repeated experiments (*P < 0.05, *t* test)

MBF1c is an extremely conserved transcriptional coactivator that plays an important role in the HSR^22,23^. Given that the overexpression of *LlWRKY39* can increase the expression of *AtMBF1c* in Arabidopsis (Fig. 4f), *Agrobacterium*-mediated transient transformation was performed with lily leaves to verify whether the same regulation mode exists in lily. The overexpression of *LlWRKY39* in lily activated the expression of *LlMBF1c* (Fig. 5a), although LlWRKY39 showed no transactivation activity in yeast cells (Supplementary Fig. S6). In addition, *LlMBF1c* and *LlWRKY39* shared similar expression patterns under HS (Supplementary Fig. S7). To further identify the regulatory mechanism between LlWRKY39 and *LlMBF1c*, we isolated and analyzed the promoter of *LlMBF1c*. The *LlMBF1c* promoter contained various *cis*-elements, such as drought responsiveness elements (MYB2AT); light responsiveness elements (I-BOX and GATA-BOX); CCAAT-BOX, the binding site of the WRKY TF family (W-box); and others (Supplementary Table S2). Then, we conducted a yeast one-hybrid assay to confirm that LlWRKY39 binds to the W-box element in the promoter region (–500 to –486 bp) of *LlMBF1c*. The yeast cells cotransformed with LlWRKY39 and the W-box element of the *LlMBF1c* promoter survived on SD medium without Leu, Trp, and His (SD-LWH), even in the presence of 75 mM 3-amino-1,2,4-triazole (3-AT), which implies that LlWRKY39 has a high affinity for the W-box element (GTCAA). However, when the GTCAA sequence was mutated to TTCAC, LlWRKY39 failed to bind to it (Fig. 5c), implying that the G and A residues in GTCAA are essential for the recognition and combination of LlWRKY39. To further verify these results, we performed an electrophoretic mobility shift assay (EMSA). As shown in Fig. 5e, the LlWRKY39-HIS complex bound to the W-box element in the *LlMBF1c* promoter and produced a mobility shift, which implies that LlWRKY39 binds to the *LlMBF1c* promoter via the W-box to regulate its expression. The effector-reporter assay showed that the *N. benthamiana* leaves cotransformed with control combinations emitted a very low LUC signal, but the signal increased significantly after cotransformation with *LlWRKY39* and *proLlMBF1c*-LUC, and the ratio of LUC/REN was also significantly higher than that of the control (Fig. 5g, h). Therefore, these results suggest that LlWRKY39 can activate the expression of *LlMBF1c* and that this activation may be achieved by directly binding the *LlMBF1c* promoter.

### LlCaM3 negatively affects LlWRKY39 in the transactivation of *LlMBF1c*

**Fig. 6:**
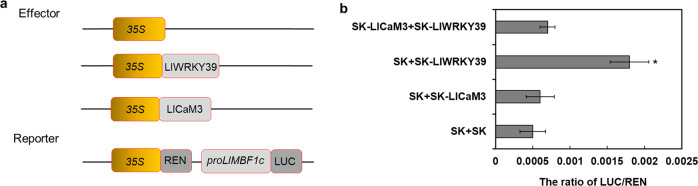
Effect of LlCaM3 on the activation activity of LlWRKY39 at the promoter of *LlMBF1c* in *N. benthamiana* leaves according to a dual-luciferase reporter assay. **a** The construction of the effect vectors and reporter vector. **b** Ratio of LUC/REN. Mixed bacterial solutions of effectors and reporter cultures (3:1). Each bar indicates the mean ± SD of three repeated experiments (*P < 0.05, *t* test)

To understand how the LlWRKY39–LlCaM3 interaction affects the function of LlWRKY39, we performed a dual-luciferase reporter assay. The ratio of LUC/REN when coexpressing *LlWRKY39* together with *LlCaM3* was significantly lower than that when only *LlWRKY39* was expressed (Fig. 6b), which suggests that the LlWRKY39-LlCaM3 interaction represses the activation ability of LlWRKY39 for its target genes.

## Discussion

Calcium, a universal secondary messenger, is one of the main signal transducers and regulators in plant cells^44–47^. The transient changes in cytosolic Ca^2+^ concentration are called Ca^2+^ signatures, and Ca^2+^ sensors respond to these changes by activating or inactivating target proteins to participate in specific biochemical processes and regulate gene expression^48^. Most Ca^2+^ sensors are proteins with one or more EF-hands that have Ca^2+^-binding helix-turn-helix structures^49^. Ca^2+^ sensors with EF-hands are roughly classified into two groups: sensor responders and sensor relays^50,51^. CaMs are a group of well-characterized Ca^2+^ sensor relays that do not have any functional domains; however, when they bind to Ca^2+^, they can activate or inactivate interacting proteins by relaying the signal^52^. AtWRKY7 was the first WRKY TF to be reported to interact with CaM in a Ca^2+^-dependent manner by binding a short conserved motif (VSSFK[K/R]VISLL in the C-region), which is also called a CBD domain^53^. AtWRKY7 belongs to the WRKY group IId subfamily, and all members of this subfamily interact with Ca^2+^/CaM in Arabidopsis^53^. Here, we identified a new WRKY IId protein, LlWRKY39, from lily, which has a similar primary motif (HARF:RTGHARFRR[A/G]P) that is the distinctive characteristic of WRKY group IId TFs^28^. Additionally, LlCaM3, which is reportedly associated with HS in lily^39^, interacts with LlWRKY39 by binding the CBD in a Ca^2+^-dependent manner (Figs. 2 and 3). Ca^2+^-dependent CBD motifs are normally grouped into two categories, namely, motifs 1-5-10 and 1-8-14, the numbers of which imply the positions of conserved hydrophobic residues^54^. The CBD of AtWRKY7 does not belong to either of the two classical CBD motifs; however, AtWRKY7 is a Ca^2+^-dependent CBP^53^. LlWRKY39 may be similar to AtWRKY7, with a CBD that is not consistent with either of the two major classes (Fig. 2d). In this study, the interaction between LlWRKY39 and LlCaM3 depends on the CBD in the N-region of LlWRKY39, although it is not completely consistent with the CBD of the WRKY IId TFs in Arabidopsis (Fig. 2c, d), which suggests that the basic CBD can interact with the CaM.

In the present study, the induction of *LlWRKY39* expression by HS implies that LlWRKY39 might participate in the regulation of HSR (Fig. 1d). Therefore, we identified the function of LlWRKY39 in the HSR process. The overexpression of *LlWRKY39* increased the thermotolerance of lily and Arabidopsis (Fig. 4b, d, e). These results were consistent with those of previous studies on *AtWRKY39* in Arabidopsis, in which the overexpressing plants showed greater thermotolerance during both seed germination and seedling growth than *wrky39* mutants or wild-type plants under HS^34^. In the current study, some of the HS-responsive genes, namely, *AtHSFA1*, *AtHSFA2*, *AtDREB2A*, *AtMBF1c*, *AtHSP101*, and *AtGolS1*, were significantly upregulated in the transgenic lines (Fig. 4f). Their elevated expression may contribute to an increase in thermotolerance since all of these genes play positive roles in thermotolerance^22,34,55–57^. Notably, the basal expression of *AtWRKY39* was also upregulated in the transgenic lines, possibly because of the self-activation of endogenous *AtWRKY39*. W-box elements in the promoters of stress-induced genes and *WRKY* genes indicate that WRKY TFs can also be self-regulated, e.g., *WRKY33*. The expression level of *AtWRKY33* is usually low in healthy plants, but when plants are exposed to environmental stimuli, activated *AtWRKY33* induces its own expression to generate a feedback mechanism for the rapid and strong induction of AtWRKY33 target genes that respond to biotic or abiotic stresses^58–60^. Nevertheless, HSFB2A, a transcriptional repressor^40^, is also induced, which is speculated to be a mechanism that maintains the balance in the HSR. In addition, although *AtHSFA1*, *AtHSFA2*, and *AtDREB2A* were induced in the transgenic plants, the expression of their target genes^41,43^, *AtHSP70*, *AtAPX1*, *AtAPX2*, and *AtHSFA3*, was not affected (Fig. 4f), which also suggests that another negative pathway exists in the LlWRKY39-mediated HSR to sustain the balance in the HSR. Among the detected genes, an important MBF1, *AtMBF1c*, was significantly induced in transgenic Arabidopsis. MBF1 proteins are highly conserved transcriptional coactivators that can build bridges between TFs and transcriptional regulation machinery^22,61–63^. *MBF1c* is a critical heat-response regulator in the HSR and functions upstream of SA, trehalose, and ethylene signaling to participate in the establishment of thermotolerance^22,23^. The homolog of *MBF1c* in lily, *LlMBF1c*, can be activated by LlWRKY39 by directly binding the W-box element in the prompter of *LlMBF1c* (Fig. 5), which suggested that LlWRKY39 might be an upstream regulator of *LlMBF1c*.

The proteins of the CaM-binding subgroup, AtWRKY7, AtWRKY11, and AtWRKY17, of the WRKY group IId proteins, play negative roles in the plant basal defense response^64,65^. *wrky7* mutants exhibited increased resistance to *Pseudomonas syringae 1*, whereas plants overexpressing *AtWRKY7* displayed a higher sensitivity to the pathogen by repressing the expression of SA-regulated defense genes or repressing weakly activated jasmonic acid (JA) signaling^64^. Similarly, *wrky11* mutants showed increased resistance to *P. syringae 1*, and *wrky11* and *wrky17* double mutants exhibited a higher increase in resistance, which was due to the decreased JA levels and the downregulation of JA-responsive genes^65^. In contrast to previous studies, LlWRKY39 activated the expression of the downstream gene *LlMBF1c*. Additionally, the interaction between LlWRKY39 and LlCaM3 repressed the expression of *LlMBF1c* activated by LlWRKY39 (Fig. 6), suggesting that Ca^2+^/CaM played an important role in inhibiting excessive activation of LlWRKY39-mediated thermotolerance to sustain balance in the HSR. Additional examples show that MBF1 proteins can reduce tolerance to stresses, although MBF1 transcription cofactors often play positive roles against many stresses. For example, in pepper, *CaMBF1* transcript levels are dramatically decreased in response to salt or cold stress, which suggests that MBF1 family factors have a negative effect on the stress response^66^. In addition, *AtMBF1* genes can relieve abscisic acid (ABA)-dependent inhibition of germination, which implies that MBF1 proteins negatively regulate the ABA-dependent response to some extent^19^. ABA signaling can also be involved in the HSR^67,68^. In this study, the interaction between LlCaM3 and LlWRKY39 weakened the transcriptional activity of *LlMBF1c* activated by LlWRKY39 to a certain extent, suggesting that this feedback regulation balances the HSR to prevent the damage caused by excessive activation responses. There may be a similar regulatory mechanism in the downstream genes of *LlMBF1c* because some genes that negatively regulate thermotolerance in transgenic lines were upregulated, e.g., *HSFB2A*, and the expression of downstream HSP genes was not changed, e.g., *HSP70*, implying the existence of negative feedback regulation in the LlWRKY39-mediated HSR.

**Fig. 7:**
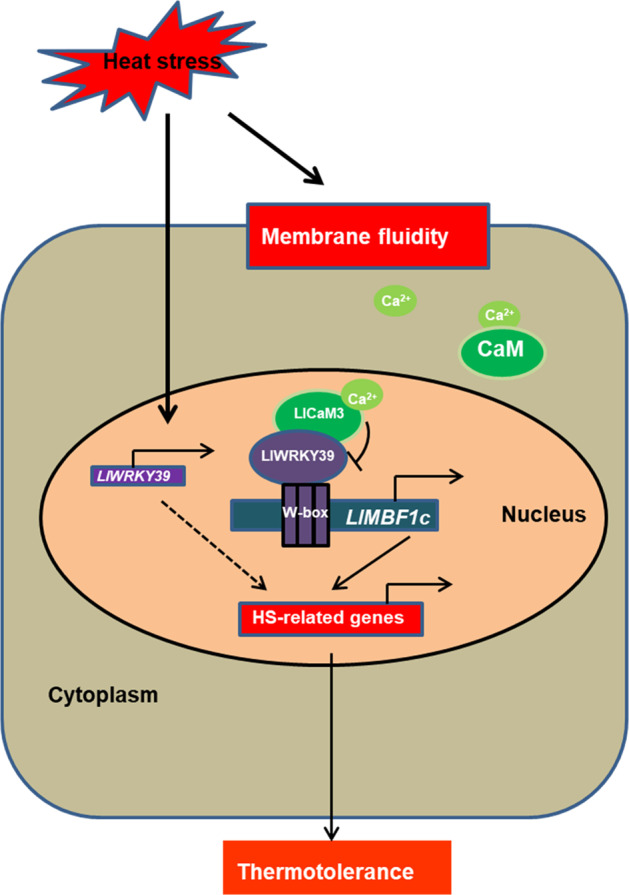
A proposed working model of LlWRKY39-mediated HSR under HS. When lily suffers from high temperature, *LlWRKY39* is rapidly induced, and its protein directly binds to the W-box element on the promoter of *LlMBF1c* to activate its expression. The feedback regulation mechanism also begins to respond, and Ca^2+^ flows into the cell to bind and activate LlCaM3; then, LlCaM3 interacts with LlWRKY39 to inhibit its activation-inducing function promoting the expression of downstream genes, e.g., *LlMBF1c*, which might contribute to the prevention of the side effects caused by excessive activation to sustain balance in the HSR

In conclusion, LlWRKY39 may act as a downstream component of the CaM-mediated calcium signaling pathway that lies upstream of *LlMBF1c* in the HSR. Considering these findings, we propose a simplified working model that may shed light on the mechanisms of the LlWRKY39-mediated HSR (Fig. 7). When lily is exposed to high temperature, LlWRKY39 is rapidly induced, which directly activates the expression of *LlMBF1c* and participates in the establishment of the HSR. Simultaneously, the feedback regulation mechanism also begins to respond, and Ca^2+^ enters the cell to activate LlCaM3, which upon interaction with LlWRKY39, can contribute to the prevention of the side effects caused by excessive activation to sustain balance in the HSR.

## Materials and methods

### Plant materials and growth conditions

The lily hybrid *Lilium longiflorum* ‘white heaven’ used in this experiment was cultured on Murashige and Skoog (MS) medium. *Arabidopsis thaliana* (Col-0) and *N. benthamiana* were grown in potting medium. All plant materials were cultured in a growth room at 22 °C with a 16-h photoperiod.

### Molecular cloning and sequence analysis of *LlWRKY39*

Total RNA was extracted from the leaves of ‘white heaven’ with TRIzol reagent according to the manufacturer’s instructions (Invitrogen, USA). cDNA was biosynthesized by a reverse transcription system (TaKaRa, Japan). Primers for gene cloning are listed in Supplementary Table S3. The physicochemical properties of LlWRKY39 were estimated by EXPASY (http://web.expasy.org/compute_pi/, default setting).

### Protein interaction assays

In the BIFC assay, the ORFs without stop codons of *LlWRKY39* and *LlCaM3* were amplified and inserted into pSPYCE (M) and pSPYNE173 vectors using a recombinant ligase (Vazyme, Nanjing, China). The reconstructed vectors and empty vectors were transformed into *Agrobacterium tumefaciens* strain GV3101. The combination of bacterial solutions (YCE:YNE:P19 = 3:3:1) was coinfiltrated into the leaves of *N*. *benthamiana*. After 48 h, a confocal laser-scanning microscope (LSM800, Zeiss, Germany) was used to observe the fluorescence signal. In the FLC assay, the ORFs without stop codons of *LlWRKY39* and *LlCaM3* were cloned into pCAMBIA1300-nLUC vectors, and the ORFs with stop codons of *LlWRKY39* and *LlCaM3* were cloned into pCAMBIA1300-cLUC vectors using recombinant ligase (Vazyme, Nanjing, China). The reconstructed vectors were transformed into *A. tumefaciens* strain GV3101. The corresponding combination of bacterial solutions (cLUC:nLUC = 1:1) was infiltrated into the leaves of *N*. *benthamiana*. After 48 h, the fluorescence signal was observed with a luminometer (PIXIS1024B, China). For a yeast two-hybrid assay, the split-ubiquitin vectors of pPR3-N and pBT3-STE were used. The ORF of *LlCaM3* was inserted into pPR3-N, and the fragments (1 to 60; 1 to 45) of *LlWRKY39* were cloned into pBT3-STE. The empty vectors pPR3-N and pBT3-STE were used as negative controls. The corresponding plasmids were cotransformed into yeast strain NMY51. The interaction was identified by spot assay on Leu-, Trp-, His-, and Ade-deficient SD medium with 3-AT (20 mM). All the primers used for vector construction are shown in Supplementary Table S3.

### CaCl_2_ and EGTA treatment

The bacterial solutions (*LlWRKY39*-*cLUC* with *LlCaM3*-*nLUC* and *LlWRKY39*-*nLUC* with *LlCaM3*-*cLUC*) were collected by centrifugation and resuspended in buffer (10 mM MgCl_2_, 200 mM acetosyringone (AS), 10 mM 2-morpholino ethanesulfonic acid (MES), pH 5.6) to a final OD_600_ of 1.0. Then, the resuspended bacterial solutions were placed in the dark for 3 h. To induce transient expression, 1-cm diameter samples were cut from *N. benthamiana* leaves with a puncher. Then, the samples were placed into the bacterial suspension and infiltrated under vacuum for 10–15 min until the samples became transparent. After the vacuum was released, the samples were placed on medium (0.4% agar) in the dark. After 24 h, the samples were transferred to medium (0.4% agar) with CaCl_2_ (20 mM) or EGTA (20 mM) and incubated for 24 h. The samples on medium (0.4% agar) were used as controls. The fluorescence signal was observed with a luminometer (PIXIS1024B, China). The LUC activity measurement was performed using Andor Solis v15 software as described in a previous study^69^.

### Subcellular localization of LlWRKY39

The ORF without the stop codon of *LlWRKY39* was inserted into a pCAMBIA1300-GFP vector to generate a fusion protein (LlWRKY39-GFP). The primers used for vector construction are shown in Supplementary Table S3. The empty vector pCAMBIA1300-GFP was used as the control. The plasmids pCAMBIA1300-GFP and pCAMBIA1300-LlWRKY39-GFP were transformed into *A. tumefaciens* strain GV3101, and then, the bacterial solution was infiltrated into *N. benthamiana*. A confocal laser-scanning microscope (LSM800, Zeiss, Germany) was used to observe the fluorescence.

### Transactivation activity assay of LlWRKY39 in yeast

The ORF of *LlWRKY39* was inserted into the pGBKT7 vector using specific primers (Supplementary Table S3). The pGBKT7 vector, pGBKT7-GAL4, and pGBKT7-LlWRKY39 were transformed into yeast strain AH109. The transformed yeast cells were incubated on SD medium lacking Trp at 30 °C for 3 days. Positive clones were selected on SD medium (lacking Trp and His) containing 3-AT at 30 °C for 3 days.

### Heat stress treatment of lily

To detect gene expression levels under HS, one-month-old ‘white heaven’ plants were exposed to 37 °C for 0, 0.5, 1, 3, 6, and 12 h. Heat treatment was applied in a temperature incubator (GZL-P80-A, Nanjing, China) without light. Samples of leaves were harvested for qRT-PCR analysis after heat treatment.

### Transient transformation in lily leaves

The bacterial solutions of pCAMBIA1300-LlWRKY39 and pCAMBIA1300 (control) were collected by centrifugation and resuspended in the same buffer as described for the CaCl_2_ and EGTA treatment. The resuspended bacterial solutions were placed in the dark for 5 h and infiltrated into the leaves of ‘white heaven’. After 72 h, the infiltrated leaves were harvested for qRT-PCR analysis.

### Gene expression analysis in lily

Total RNA was extracted using TRIzol as described above. A HiScript II Kit with gDNA Eraser (Vazyme, Nanjing, China) was used for cDNA biosynthesis with an oligo dT primer. qRT-PCR was performed using a 20-μL reaction system. Lily *18S rRNA* was used as the endogenous gene. Three independent technical replicates were performed for each of three biological replicates. The relative expression level was calculated with the 2^–∆∆Ct^ method^70,71^. The primers for qRT-PCR are shown in Supplementary Table S3.

### Isolation and analysis of promoter sequences

The genomic DNA of ‘white heaven’ was extracted using a plant DNA extraction kit (Zomanbio, Beijing, China) following the manufacturer’s instructions. The promoter was isolated using hi-TAIL PCR^72^. PLACE databases (http://www.dna.affrc.go.jp/PLACE/) were used to analyze the *cis*-elements in the promoters.

### Promoter activity analysis of *LlWRKY39*

The isolated 737-bp fragment of the *LlWRKY39* promoter was fused to a pGreenII-0800-LUC vector using a recombinant ligase (Vazyme, Nanjing, China). The constructed vector and an empty vector (control) were transformed into *A. tumefaciens* strain GV3101 (pSoup), which was used to transform *N. benthamiana* leaves. After 48 h, one-half of the leaves with infiltrated material were treated at 37 °C for 2 h, and allowed to recover from HS at 22 °C for 12 h; then, the fluorescence signal was observed with a luminometer (PIXIS1024B, China). A luciferase reporter assay system (Promega, USA) was used to measure the activity of firefly luciferase (LUC) and *Renilla* luciferase (REN). The primers for vector construction are shown in Supplementary Table S3.

### Plant transformation and generation of transgenic lines

It is difficult to obtain lily transgenic plants since a stable genetic transformation system of lily has not yet been established. Therefore, the model plant *A. thaliana* (Col-0) was used to verify the biological function of *LlWRKY39* under HS. The bacterial solution of pCAMBIA1300-LlWRKY39 was collected by centrifugation and resuspended in a sucrose solution (5%). Arabidopsis plant genetic transformation was performed according to the floral dip method^73^. The harvested seeds were selected on MS medium containing 30 mg/L hygromycin until the T3 generation. One-week-old seedlings were collected to extract RNA for RT-PCR identification of the transgenic lines.

### **Phenotypic analysis and heat stress responsive gene expression in transgenic plants**

Seeds of three transgenic lines (OE-2, OE-4, OE-5) and Col-0 were sown on the same MS medium, and after incubation, they were placed into the culture room at 4 °C for 3 days. To compare the thermotolerance of the transgenic line and wild-type (WT) plants, one-week-old seedlings were treated with two HS tests. One was a BT-test in which seedlings were directly treated at 45 °C (shown in Fig. 4d), whereas the other was an AT-test in which seedlings were first treated at 37 °C for 1 h, recovered for 2 h at 22 °C, and then treated again at 45 °C (shown in Fig. 4e). After these treatments, the plants were placed in the culture room for a 7-day recovery, and the phenotypes and survival rates of the seedlings were recorded. One-week-old seedlings of the transgenic and WT plants were collected to determine the expression levels of heat-related genes: *AtHSFA1* (*AT4G17750*), *AtHSFA2* (*AT2G26150*), *AtHSFA3* (*AT5G03720*), *AtHSFB2A* (*AT5G62020*), *AtDREB2A* (*AT2G40340*), *AtWRKY39* (*AT3G04670*), *AtAPX2* (*AT3G09640*), *AtAPX1* (*AT1G07890*), *AtGolS1* (*AT2G47180*), *AtMBF1c* (*AT3G24500*), *AtHSP70* (*AT3G12580*), and *AtHSP101* (*AT1G74310*). *AtActin2* (*AT3G18780*) was used as the endogenous gene. The primers used for qRT-PCR are shown in Supplementary Table S3.

### Yeast one-hybrid assay

A fragment (-500 to -486) of the *LlMBF1c* promoter with three repeats was cloned into pHis2.1 to obtain the pHis2.1–3 × W-box; two sites of this fragment (Fig. 5b) were mutated and cloned into pHis2.1 to generate pHis2.1–3 × mW-box. *LlWRKY39* was amplified and inserted into the pGADT7 vector. Different combinations of plasmids were cotransformed into yeast strain Y187 to identify positive clones. SD medium lacking Trp, Leu, and His was used to detect possible interactions. The primers used for vector construction are listed in Supplementary Table S3.

### Luciferase reporter assay

The 836-bp fragment of the *LlMBF1c* promoter was cloned into pGreenII-0800-LUC to obtain the reporter vector. The ORF of *LlWRKY39* was cloned into a pGreenII-62-SK vector to generate the effector vector. These vectors were transformed into *A. tumefaciens* strain GV3101 (pSoup). The mixed bacterial solution of the TF and promoter cultures (2:1) for the induction analysis was used to infiltrate *N. benthamiana* leaves. After 48 h, the LUC fluorescence of the infiltrated leaves was detected, and LUC and REN activities were measured using luciferase reporter assay reagents (Promega) as described by a previous study^74^. Three replicate experiments were used for statistical analysis by Student’s *t* test.

### Electrophoretic mobility shift assay (EMSA)

An EMSA was performed using the Light Shift Chemiluminescent EMSA kit (Thermo Fisher, New York, USA) according to the manufacturer’s protocol. The biotinylated probes for the EMSAs were synthesized by TSINGKE Biological Technology (Nanjing). The recombinant proteins were purified as described in a previous study^75^. The samples were loaded onto a prerun native 4% polyacrylamide gel with TBE buffer as the electrolyte. After electroblotting onto a nylon membrane (Millipore, Darmstadt, Germany) and UV cross-linking for 2 min, the membrane was incubated in blocking buffer for 15 min and rinsed in washing buffer for 20 min. Finally, a CCD camera was used to visualize the signals in the membrane.

### Measurement of relative ion leakage

*Agrobacterium*-mediated transformation was performed as described above, and then ‘white heaven’ plants were treated with HS at 42 °C for 2 h. The leaves were harvested to measure ion leakage (percentage) according to a previously described method^6^.

### Dual-luciferase reporter assay

The ORFs of *LlWRKY39* and *LlCaM3* were cloned into pGreenII-62-SK^76^ for use as effector vectors. A fragment of the *LlMBF1c* promoter was inserted into pGreenII-0800-LUC^76^, which was used as the reporter vector. These vectors were transformed into *A. tumefaciens* strain GV3101 (pSoup). Mixed bacterial solutions of effector and reporter cultures (3:1) were used to infiltrate the leaves of *N. benthamiana*. The LUC and REN activity levels were measured as described above. The primers for the vector construction are shown in Supplementary Table S3.

## Supplementary information

Supplementary Figures

Supplementary Tables
