# Supplementary material for: LlWRKY39 is involved in thermotolerance by activating LlMBF1c and interacting with LlCaM3 in lily (Lilium longiflorum)
Source: Hortic Res. 2021 Feb 4;8:36. doi: 10.1038/s41438-021-00473-7 (PMC7862462; doi:10.1038/s41438-021-00473-7)
Supplement: Supplementary file 1 — Supplementary Figures [file 41438_2021_473_MOESM1_ESM.docx]

**Supplementary information**

This file contains Supplementary Figures S1-S7.


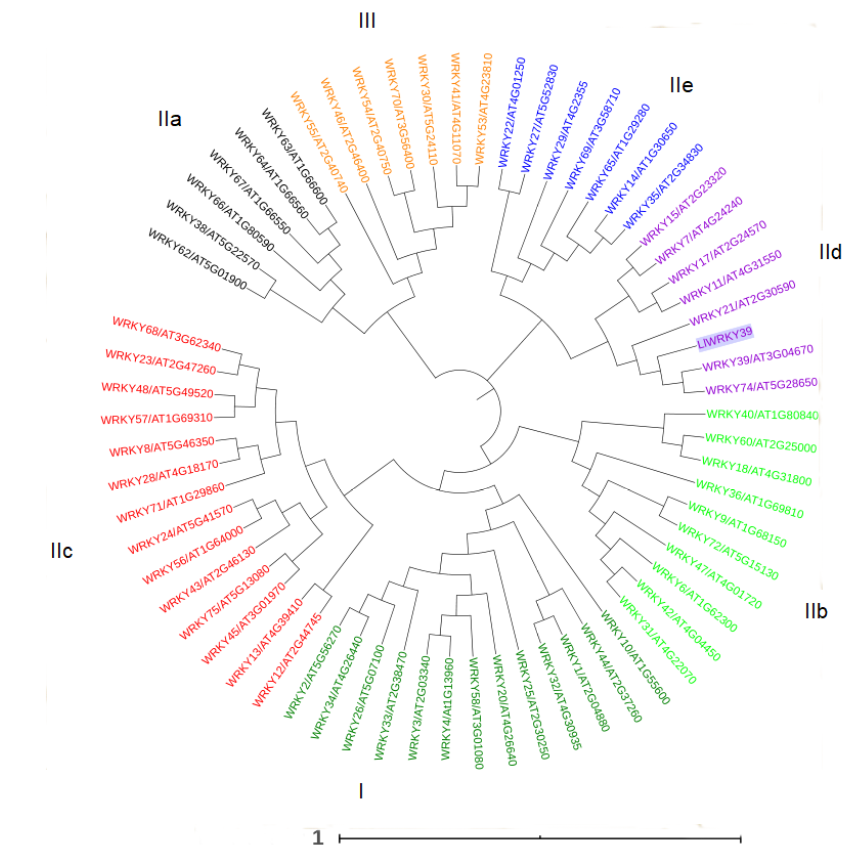


**Supplementary Fig. S1 Phylogenetic relationship of LlWRKY39 with all WRKYs proteins from Arabidopsis.** The Phylogenetic tree was constructed by iTOL online tool ([https://itol.embl.de/tree/) with](https://itol.embl.de/tree/)%20with) the default parameters. The amino acid sequences of the Arabidopsis WRKYs were downloaded from TAIR (<https://www.arabidopsis.org/>).


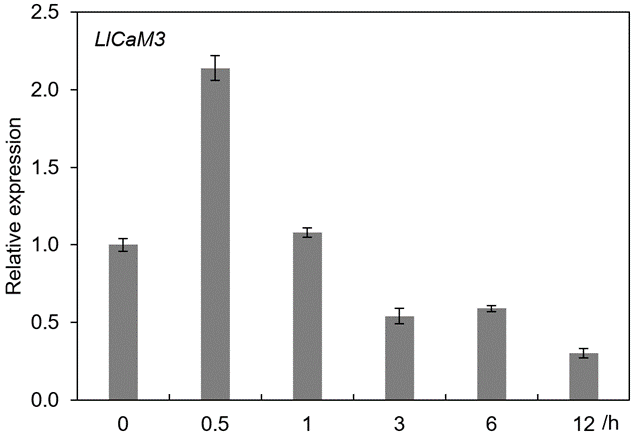


**Supplemental Fig. S2 Detection of expression level of the gene *LlCaM3* under HS by qRT-PCR. Leaf samples were collected for qRT-PCR.** Lily *18S rRNA* was used as the normalization control. Data represent means ± SD of three independent experiments.


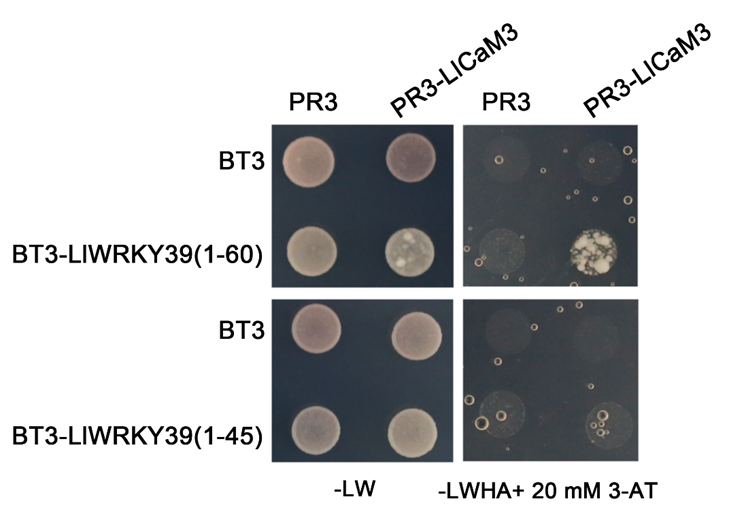


**Supplementary Fig. S3 Yeast two-hybrid (Y2H) assay.** Protein–protein interactions were examined by cell growth on selective media lacking Leu, Trp, His, Ade (-LWHA), but containing 3-AT (20 mM). At least three yeast cells were tested in each combination; one representative is shown.


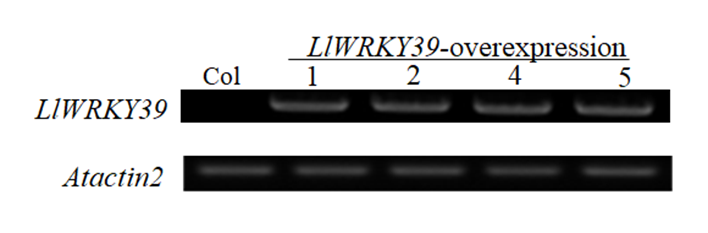


**Supplementary Fig. S4** **Molecular analysis of *LlWRKY39* transgenic Arabidopsis lines.** One-week-old seedlings were used to detect the expression of *LlWRKY39* in transgenic Arabidopsis lines by RT-PCR. *AtActin2* was used as an endogenous gene.


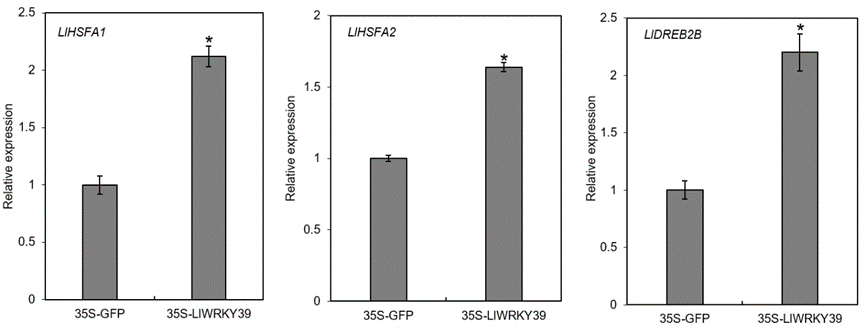


**Supplementary Fig. S5 Detection of expression level of genes *LlHSFA1*, *LlHSFA2* and *LlDREB2B* in overexpressed *LlWRKY39* lily by qRT-PCR.** Lily *18S rRNA* was used as the control. Data represent means ± SD of three independent experiments (*t*-test; *, P < 0.05).


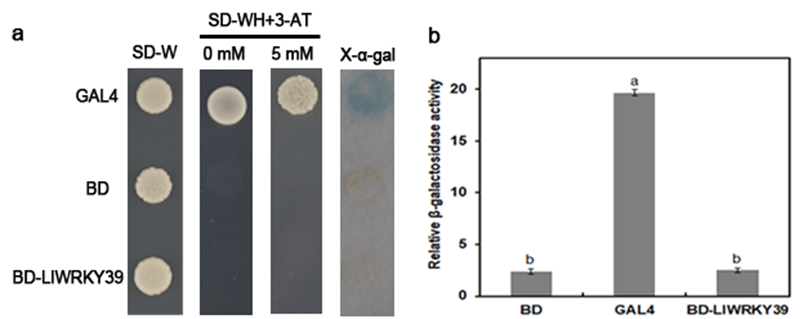


**Supplementary Fig. S6 Transactivation assay of LlWRKY39.** **a** Transactivation activity assay in yeast cells. GAL4 and BD were the respective positive and negative controls. SD medium lacking Trp (SD-W) was used to detect transformation; SD medium lacking Trp and His (SD-WH) with or without 3-AT was used to examine the growth of transformants; and X-α-gal staining was used to detect *β*-galactosidase activity of transformed yeast cells. **b** Measurement of *β*-galactosidase activity. Bars are the mean ± SD of three replicated experiments. Different letters are indicated by Student–Newman–Keuls test.


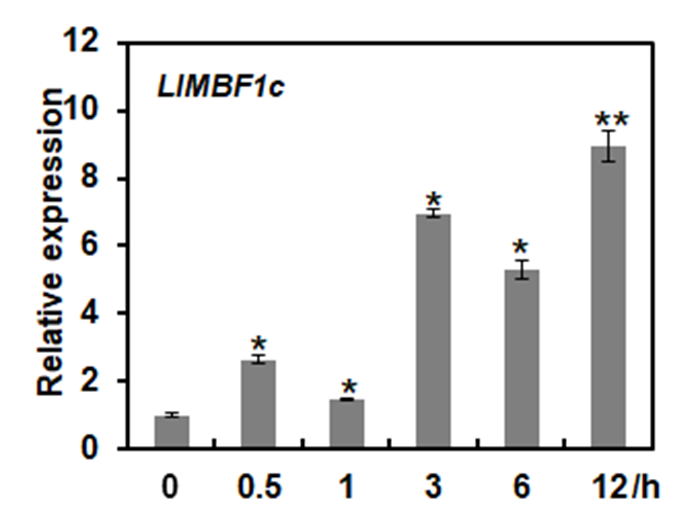


**Supplementary Fig. S7 Expression levels of *LlMBF1c* in lily leaves with 37 ℃ treatment at different lengths of time.** Bars represent the mean ±SD of three repeated experiments (**P*<0.05, and ***P* < 0.01, Student’s *t*-test)
