# Supplementary material for: LlWRKY39 is involved in thermotolerance by activating LlMBF1c and interacting with LlCaM3 in lily (Lilium longiflorum)
Source: Hortic Res. 2021 Feb 4;8:36. doi: 10.1038/s41438-021-00473-7 (PMC7862462; doi:10.1038/s41438-021-00473-7)
Supplement: Supplementary file 2 — Supplementary Tables [file 41438_2021_473_MOESM2_ESM.pdf]

Supplementary information

This file contains Supplementary Tables S1-S3.

Supplementary Table. S1 Predication of cis-element of *LlWRKY39*

Element name	Function	Core sequence	Number
CAATBOX	enhancer of transcription	CAAT	7
TATABOX	transcription initiation site	TTATTT	2
I-BOX	light response element	GATAA	2
MYB1AT	binding site of MYB and MYC transcription factors involved in ABA	WAACCA	1
GATA-BOX	light response element	GATA	4
CCAAT-BOX	heat stress related elements	CCAAT	4
W-BOX	binding sites for WRKY	(T)(T)TGAC(C/T)	2
AAAG-element	binding sites of transcription factor Dof	AAAG	10
TAAAG-element	binding sites of transcription factor Dof that is involved in regulating the expression of specific genes in guard cells	TAAAG	2
POLASIG1	seed germination related elements	AATAA	1
POLLEN1	pollen specific expression of the necessary elements	AGAAA	8
GARE	the gibberellin-responsive element was found in the promoter of the cysteine protease gene in the aleurone layer of barley, which was involved in the inhibition of the expression of the α amylase gene by glucose in rice embryo	TTTTTTCC	1
PYRIMIDINEBOX	gibberellin response element is involved in inhibiting the expression of the α amylase gene by glucose in rice embryo	CCTTTT	2
ROOT element	root expression-related elements	ATATT	2

Supplementary Table. S2 Predication of cis-element of *LlMBF1c*

Element name	Function	Core sequence	Number
CAATBOX 1	enhancer of transcription	CAAT	6
W-BOX	binding sites for WRKY	(T)(T)TGAC(C/T)	12
MYB2AT	binding site for ATMYB2 that are responsive to drought stress in Arabidopsis	TAACTG	1
TATABOX	transcription initiation site	TTATTT	2
CCAAT-BOX	heat stress related elements	CCAAT	2
I-BOX	light response element	GATAA	1
GATA-BOX	light response element	GATA	8
TAAAG-element	binding site of zinc finger transcription factor Dof, which is involved in regulating the expression of specific genes in guard cells	TAAAG	3
POLASIG1	seed germination related elements	AATAA	1
MYB1AT	binding site of MYB and MYC transcription factors involved in ABA	WAACCA	1
MYBCORE	binding sites of MYB transcription factor in the epidermis of petunia petals	CNGTTR	2
ROOT element	root expression-related elements	ATATT	4
E-BOX	elements in the promoter of the storage protein gene	CANNTG	2

Supplementary Table. S3 The primers used in this article	
Name	Sequences(5'to3')
**Primers for gene cloning**	
LlWRKY39-F	ATGGAAGAGGTTGACTCAGCCA
LlWRKY39-R	TTATGCATGGGCAGATTGTGTT
**Primers for Subcellular localization**	
LlWRKY39- Xba I-*F*	ACTCTAGAATGGAAGAGGTTGACTCAGCCA
LlWRKY39- BamH I-*R*	CGGGATCCTTATGCATGGGCAGATTGTGTT
**Primers for transactivation assay**	
BD-LlWRKY39-EcoR I-*F*	CGGAATTCATGGAAGAGGTTGACTCAGCCA
BD-LlWRKY39-BamH I-*R*	CGGGATCCTTATGCATGGGCAGATTGTGTT
**Primers for overexpression in *Arabidopsis***	
LlWRKY39- Xba I-*F*	ACTCTAGAATGGAAGAGGTTGACTCAGCCA
LlWRKY39- BamH I-*R*	CGGGATCCTTATGCATGGGCAGATTGTGTT
**Primers for RT-PCR**	
RT-LlWRKY39-F	ATCACCATGATGAGCTGCAGAGCAG
RT-LlWRKY39-R	GCAGATTGTGTTAGTAGCTTGGGGT
**Primers for qRT-PCR**	
qRT-AtMBF1c-F	AGCAGATACCCAGGAGCAGT
qRT-AtMBF1c-R	TTCGGATCGCGTAGGTCTTG
qRT-AtAPX1-F	ATTCAGATGCCCAGAAGTCTTGTTCC
qRT-AtAPX1-R	ACCACCGATCCAGACACTGTACTTCC
qRT-AtHSP101-F	ATGTTGGTCACGAGGAAGGT
qRT-AtHSP101-R	TGAAATCGACTGTCCTGCCT
qRT-AtHSP70-F	GAGAGGGCACGAACAAAGGA
qRT-AtHSP70-R	GTCCTCAGCCGACACATTCA
qRT-AtAPX2-F	TCAGGATTCGAGGGTGCATG
qRT-AtAPX2-R	AAGGCATCCTCATCTGCAGC
qRT-AtGolS1-F	AGCCACCGGCTCTTTACTTC
qRT-AtGolS1-R	GTTCAGCGAAAGGAGTCGGA
AtActin2-F	TCCCTCAGCACATTCCAGCAGAT
AtActin2-R	AACGATTCCTGGACCTGCCTCATC
qRT-AtHSFB2A-F	CTCTCAGCCTGCTATGGCCGCGGCTG
qRT-AtHSFB2A-F	GTGCAGTGGTGCAGCTCGTTGTCCTCTG
qRT-AtDREB2A-F	CAGTGTTGCCAACGGTTCAT
qRT-AtDREB2A-R	AAACGGAGGTATTCCGTAGTTGAG
qRT-AtHSFA2-F	GTGTTGAGGTTGGGCAATACG
qRT-AtHSFA2-R	TTGCTGTTGCCTCAACCTAACTAC
qRT-AtHSFA3-F	CCAAGGAATTTCAAACACAACA
qRT-AtHSFA3-R	TCGTTAGCGAATTCCCACTT
qRT-AtWRKY39-F	TGCGGAAGTCGAAGCAAATGTCA
qRT-AtWRKY39-R	CGTGGATGCGGTGAACCCTTTAT
qRT-AtHSFA1a-F	CCAGATACCACAATTGACACGAGAG

qRT-AtHSFA1a-R	GGTATTCCCTCAAGCTGAATCG
qRT-LlMBF1C-F	GCTGATTAACGAGCGGGTGC
qRT-LlMBF1C-R	CACCCGCTCCATCTTCGCAAG
qRT-LlWRKY39-F	CTTGGAGGAAATATGGGCAGAAGCC
qRT-LlWRKY39-R	GTGTTAGTAGCTTGGGGTGG
qRT-L1CaM3-F	CATGTCATGACTAACCTAGGCGAG
qRT-L1CaM3-R	CCATTAGAAATCAGCCAGCACC
qRT-L1DREB2B-F	CTTTGCAGGGAGGGAGCTTGTTCT
qRT-L1DREB2B-R	ACTAGCAGCATACTAGCCTAATCCCT
qRT-L1HSFA1-F	ATGGGAAGTGTCTATGTGGGG
qRT-L1HSFA1-R	CATTGATACTTGGCAGTTGTTGG
qRT-L1HSFA2-F	CAGACTGAGGTCGAGTTGGAAG
qRT-L1HSFA2-R	AACACAGCCCTCTTATCTTCTCG
**Primers for isolation of promoter of *LlWRKY39* and *LlMBF1c***	
LAD1	ACGATGGACTCCAGAGCGGCCGC(G/C/A)N(G/C/A)NNNGGAA
LAD2	ACGATGGACTCCAGAGCGGCCGC(G/C/T)N(G/C/T)NNNGGTT
LAD3	ACGATGGACTCCAGAGCGGCCGC(T/A/C)N(A/G/C)NNNCCAC
LAD4	ACGATGGACTCCAGAGCGGCCGC(G/C/A)(G/C/A)N(G/C/A)NNNCCAA
LAD5	ACGATGGACTCCAGAGCGGCCGC(G/C/T)(G/A/T)N(G/C/T)NNNCGGT
AC1	ACGATGGACTCCAGAG
LlWRKY39-R1	AGGAGATCTGTTGAGTGCTCTAACCCT
LlWRKY39-R2	GCCTCCCATGTTTGAGCCTGGAGGGT
LlWRKY39-R3	GGCAGCTCTGTACAGCAGCTCTGCTGGC
LlMBF1c-R1	ATTGTCACCAATTTCCCGGGGACCATCCA
LlMBF1c-R2	AGCCTGTTTCCCCGTACCTAGACAAGTCGT
LlMBF1c-R3	GACGGAGTCGGGGCTTTTTGGGACATCCTTAC
**Primers for calibration of promoter of *LlWRKY39* and *LlMBF1c***	
LlWRKY39-F1	TAATATACTGGTTCGGGTCGGCG
LlWRKY39-F2	ACCCTCTCAGGTCGGGAACTTT
LlWRKY39-R	CAGCTCTGTACAGCAGCTCTGCTGGC
LlMBF1c-F1	CGGCCGCGAGTAGGGAAACTGCGAAG
LlMBF1c-F2	ATACGGGTTACCGAAAGGGCTACTA
LlMBF1c-R	TGATGATGATAGAGAAGTCAACAAGG
**Primers for promoter activity assay of *LlWRKY39***	
LUC-LlWRKY39-Kpn I-*F*	TTGTAATACGACTCACTATAGGGCGAATTGGGTACCACCCTCTCAGGTCGGGAAC
LUC-LlWRKY39-Xho I-*R*	TTCGATATCAAGCTTATCGATACCGTCGACCTCGAGCTTGGATTTCAGCAGCTCAG

**Primers for BIFC vectors construction**	
YCE-LlWRKY39-Xba I-*F*	TCATTTGGAGAGAACACGGGGGACTCTAGAATGGAAGAGGTTGACTCAGCC
YCE-LlWRKY39-Kpn I-*R*	ATCGTATGGGTACATCCCGGGAGCGGTACCTGCATGGGCAGATTGTGTTA
YNE-LlWRKY39-Xba I-*F*	TCATTTGGAGAGAACACGGGGGACTCTAGAATGGAAGAGGTTGACTCAGCC
YNE-LlWRKY39-Kpn I-*R*	CAACTTTTGCTCCATCCCGGGAGCGGTACCTGCATGGGCAGATTGTGTTA
YCE-LlCaM3-Xba I-*F*	TCATTTGGAGAGAACACGGGGGACTCTAGAATGGCGGATCAGCTCACTGAT
YCE-LlCaM3-Kpn I-*R*	ATCGTATGGGTACATCCCGGGAGCGGTACCCTTAGCCATCATGACTTTGAC
YNE-LlCaM3-Xba I-*F*	TCATTTGGAGAGAACACGGGGGACTCTAGAATGGCGGATCAGCTCACTGAT
YNE-LlCaM3-Kpn I-*R*	CAACTTTTGCTCCATCCCGGGAGCGGTACCCTTAGCCATCATGACTTTGAC
**Primers for FLC vectors construction**	
cLUC-LlWRKY39-Kpn I-*F*	CAGATCTCGTACGCGTCCCGGGGCGGTACCATGGAAGAGGTTGACTCAGCCAGCA
cLUC-LlWRKY39-Sal I-*R*	ATGATACGAACGAAAGCTCTGCAGGTCGACTTATGCATGGGCAGATTGTGTTAGT
nLUC-LlWRKY39-Kpn I-*F*	GGAGAGAACACGGGGGACGAGCTCGGTACCATGGAAGAGGTTGACTCAGCCAGCA
nLUC-LlWRKY39-Sal I-*R*	GCCCCGGGACGCGTACGAGATCTGGTCGACTGCATGGGCAGATTGTGTTAGT
cLUC-LlCaM3-Kpn I-*F*	CAGATCTCGTACGCGTCCCGGGGCGGTACCATGGCGGATCAGCTCACTGATGAC
cLUC-LlCaM3-Sal I-*R*	ATGATACGAACGAAAGCTCTGCAGGTCGACTCACTTAGCCATCATGACTTTGAC
nLUC-LlCaM3-Kpn I-*F*	GGAGAGAACACGGGGGACGAGCTCGGTACCATGGCGGATCAGCTCACTGATGAC
nLUC-LlCaM3-Sal I-*R*	GCCCCGGGACGCGTACGAGATCTGGTCGACCTTAGCCATCATGACTTTGAC
**Primers for dual-luciferase vectors construction**	
SK-LlWRKY39-Xba I-*F*	GAGCTCCACCGCGGTGGCGGCCGCTCTAGAATGGAAGAGGTTGACTCAGCCAGCA
SK-LlWRKY39-BamH I-*R*	TGATATCGAATTCCTGCAGCCCGGGGGATCCTTATGCATGGGCAGATTGTGTTAGT
SK-LlCaM3-Xba I-*F*	GAGCTCCACCGCGGTGGCGGCCGCTCTAGAATGGCGGATCAGCTCACTGATGAC
SK-LlCaM3-BamH I-*R*	TGATATCGAATTCCTGCAGCCCGGGGGATCCTCACTTAGCCATCATGACTTTGAC

LUC-LlMBF1c-Kpn I-*F*	TTGTAATACGACTCACTATAGGGCGAATTGGGTACCCGGCCGCGAGTAGGGAAACT
LUC-LlMBF1c-Xho I-*R*	TTCGATATCAAGCTTATCGATACCGTCGACCTCGAGTGATGATGATAGAGAAGTCA
**Primers for yeast one-hybrid vectors construction**	
pHis2-ProMBF1C-EcoR I-*F*	CGGAATTCTAGTCAAATTAGTCAAATTAGTCAAATTAGTCAAATTAGTCAAATGAGCTC
*pHis2-ProMBF1C-Xho*I-*R*	GAGCTCATTTGACTAATTTGACTAATTTGACTAATTTGACTAATTTGACTAGAATTCCG
AD-LlWRKY39-Nde I-*F*	GGAATTCCATATGATGGAAGAGGTTGACTC
AD-LlWRKY39-BamH I-*R*	CGGGATCCATTTATGCATGGGCAGATTGTG
**Primers for EMSA**	
W-box-F	ACTAAAAATTATAGTCAAATTACATCATAAACTAAAAATTATAGTCAAATTACATCATAA
W-box-R	TTATGATGTAATTTGACTATAATTTTTAGTTTATGATGTAATTTGACTATAATTTTTAGT
